# *Fomitopsis officinalis*: Spatial (Pileus and Hymenophore) Metabolomic Variations Affect Functional Components and Biological Activities

**DOI:** 10.3390/antibiotics12040766

**Published:** 2023-04-16

**Authors:** Giancarlo Angeles Flores, Gaia Cusumano, Federica Ianni, Francesca Blasi, Paola Angelini, Lina Cossignani, Roberto Maria Pellegrino, Carla Emiliani, Roberto Venanzoni, Gokhan Zengin, Alessandra Acquaviva, Simonetta Cristina Di Simone, Maria Loreta Libero, Nilofar, Giustino Orlando, Luigi Menghini, Claudio Ferrante

**Affiliations:** 1 Department of Chemistry, Biology and Biotechnology, University of Perugia, 06122 Perugia, Italy; giancarlo.angelesflores@unich.it (G.A.F.); gaia.cusumano@studenti.unipg.it (G.C.); paola.angelini@unipg.it (P.A.); roberto.pellegrino@unipg.it (R.M.P.); carla.emiliani@unipg.it (C.E.); roberto.venanzoni@unipg.it (R.V.); 2 Department of Pharmaceutical Sciences, University of Perugia, 06126 Perugia, Italy; federica.ianni@unipg.it (F.I.); francesca.blasi@unipg.it (F.B.); 3 Center for Perinatal and Reproductive Medicine, Santa Maria della Misericordia University Hospital, University of Perugia, 06132 Perugia, Italy; 4 Physiology and Biochemistry Research Laboratory, Department of Biology, Science Faculty, Selcuk University, 42130 Konya, Turkey; gokhanzengin@selcuk.edu.tr; 5 Botanic Garden “Giardino dei Semplici”, Department of Pharmacy, “Gabriele d’Annunzio” University, 66100 Chieti, Italy; alessandra.acquaviva@unich.it (A.A.); simonetta.disimone@unich.it (S.C.D.S.); maria.libero@unich.it (M.L.L.); nilofar.nilofar@unich.it (N.); giustino.orlando@unich.it (G.O.); claudio.ferrante@unich.it (C.F.)

**Keywords:** *Fomitopsis officinalis*, metabolomics, phenolic compounds, antimicrobial effects, antiradical effects

## Abstract

*Fomitopsis officinalis* is a holartic polyporous mushroom that forms large fruiting bodies on old standing trees, fallen logs, or stumps. *F. officinalis* is a medicinal mushroom species that is most commonly used in traditional European medicine. In this study, we explore the spatial metabolic differences in *F. officinalis’* mushroom parts, i.e., the cap (median and apical parts) and the hymenium. Additionally, chromatographic analysis was conducted in order to unravel the composition of specialized metabolites in the hydroalcoholic mushroom extracts. The potential antifungal and bacterial effects of extracts were tested against pathogen strains of Gram+ and Gram– bacteria, and yeast, dermatophytic, and fungal-pool species. Extracts from the apical part were the richest in terms of phenolic compounds; consistent with this finding, the extracts were also the most effective antiradical and antimicrobial agents with MIC values < 100 µg/mL for most of the tested bacterial and dermatophytic species. According to these findings, *F. officinalis* extracts are valuable sources of primary and secondary metabolites, thus suggesting potential applications in the formulation of food supplements with biological properties in terms of antioxidant and antimicrobial activities.

## 1. Introduction

*Fomitopsis officinalis* (Vill.) Bondartsev and Singer (syn. *Laricifomes officinalis* (Vill.) Kotl. and Pouzar) is a holartic polyporous mushroom belonging to the *Fomitopsidaceae* family (Polyporales order) that forms large fruiting bodies on old standing trees, fallen logs, or stumps. It is a slow-growing necrotrophic parasite causing intensive brown rot in wood. Basidiomata are perennial and sometimes reach a considerable size [1,2]. *F. officinalis* is easily distinguished from other *Fomitopsis* species due to its chalky texture, and distinctive smell and taste [1,2].

The existence of this species was reported in Europe, Asia, North Africa, and North America. Western Europe (the Alps), North Asia (Siberia from the Ural Mountains to the Russian Far East), and North America are the three major distribution areas of *F. officinalis* [3,4]. This species was reported on several hosts in North America, such as *Abies* Mill., *Larix* Mill., *Picea* A. Dietr., *Pinus* L., *Pseudotsuga* Carrière, and *Tsuga* Carrière [5]. In Eurasia, it can be found mostly on *Larix* spp. and sometimes on *Pinus* spp. In Europe, it has been frequently recorded on *Larix decidua* Mill. [4]. *F. officinalis* is a medicinal mushroom species that was most commonly used in traditional European medicine [6]. This species contains molecules such as agaricinic acid and chlorinated coumarins with antibacterial activity against Gram bacteria, *Mycobacterium tuberculosis*, the *Herpes simplex* virus, *Poxviridae*, *Orthopoxvirus*, and the Type A influenza virus in birds (H5N1) and humans (H3N2) [3].

Chlorinated coumarins from mycelia and lanostane triterpenoids from basidiomes are directly responsible for antiviral or antibacterial and trypanocidal activity, respectively [3]. Among the bioactive compounds of *F. officinalis*, lanostane-type triterpenoids constitute the main group [6]. Other biological activities of *F. officinalis* extracts are anticancer [7] and anti-inflammatory [8]. Recently, one of these compounds (dehydrosulfurenic acid), specifically found in *F. officinalis*, was patented as a potential pharmaceutical treatment for ischemic stroke [9].

Metabolomics provides a quantitative and/or qualitative estimation of all low metabolites (small molecules with a molecular weight of less than 1800 Da) present in biological systems, for instance, fungi, plants, animals, and prokaryotes [10,11]. In recent years, metabolomics has emerged as an important methodology to determine the temporal and spatial correlations between metabolic cues and associated phenotypes in mushroom species [12]. A large number of studies elucidated the gamut of important metabolites in *F. officinalis* for their organoleptic properties, flavor, and nutritional and functional aspects, particularly anticancer potential [13,14]. Although the majority of studies characterized the lanostane-type triterpenoids class of secondary metabolites from *F. officinalis* [15,16,17,18], the spatial distribution of primary and secondary metabolomes in different mushroom parts is largely unknown with respect to the associated phenotypes.

In this study, we designed a nontargeted mass spectrometry (MS)-based metabolomic framework to explore the spatial metabolic differences in *F. officinalis* mushroom parts, i.e., the cap (median and apical parts) and the hymenium. Further, we established the correlation between the discriminant metabolite classes, and the specific mushroom parts (phenotypes) and bioactivities. Additionally, chromatographic analysis was conducted in order to unravel the composition of specialized metabolites in the mushroom extracts prepared with a hydroalcoholic solution. Considering the potential antifungal and antibacterial effects of extracts from edible mushrooms [11,19], the above-mentioned extracts were tested for potential growth-inhibitory effects against the pathogen strains of Gram+ and Gram- bacteria, and yeast, dermatophytic, and fungal-pool species.

## 2. Results and Discussion

### 2.1. Mushroom Identification

The exact characterization and identification of medicinal mushrooms is fundamental for exploiting their full potential in the food and pharmaceutical industries [20]. The morphological characteristics of the fruiting body of *F. officinalis* (PeruMyc 3897) correspond to those reported by Bernicchia and Gorjón [21]. The taxonomic affiliation of the mushroom strains was performed via targeting the ITS region of the ribosomal DNA. Additionally, a BLAST search confirmed that our sample belonged to *F. officinalis*, as it showed a close match with the deposited sequences of these species (Table 1).

### 2.2. Untargeted LC–MS/MS-Based Metabolomics

In this study, the metabolomic profile of *F. officinalis* was evaluated through the mass spectrometry–ultraperformance liquid chromatography (UHPLC)–QTOF method. The raw data were processed with MS-DIAL in two separate sessions, one for the Pos and one for the Neg files. The obtained data were merged into a single data matrix that reports the mass and retention time of 8335 features, and their area in the respective 9 samples (from L1 to L9).

### 2.3. Statistical Data Analysis

**Figure 1:**
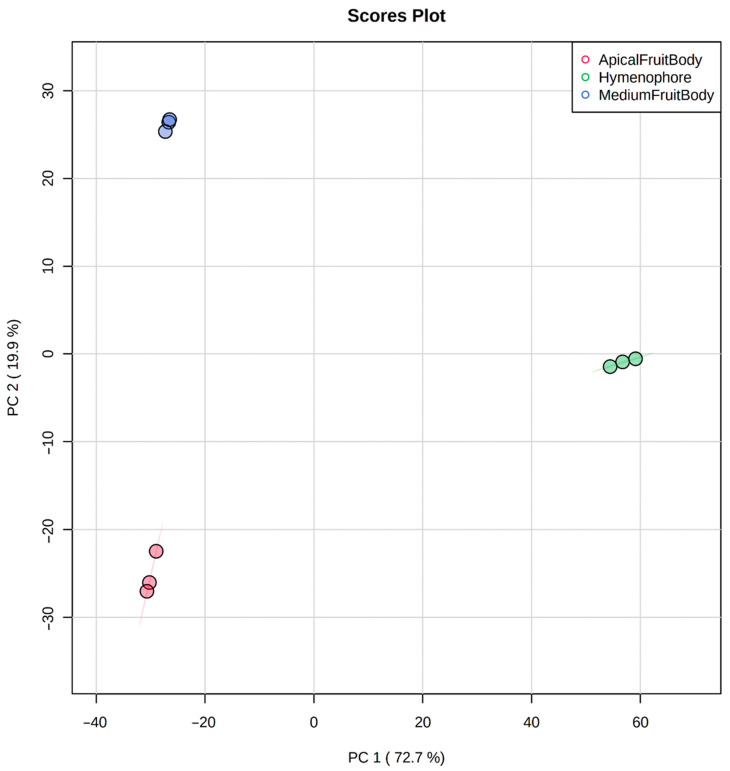
Almost 73% of the variance is explained by the first component of the PCA, where a clear separation of the hymenium from the fruiting body was observed. A 20% of the residual variance is explained by the second main component. In this case, an excellent separation of the fruiting body was observed in the apical and middle parts.

**Figure 2:**
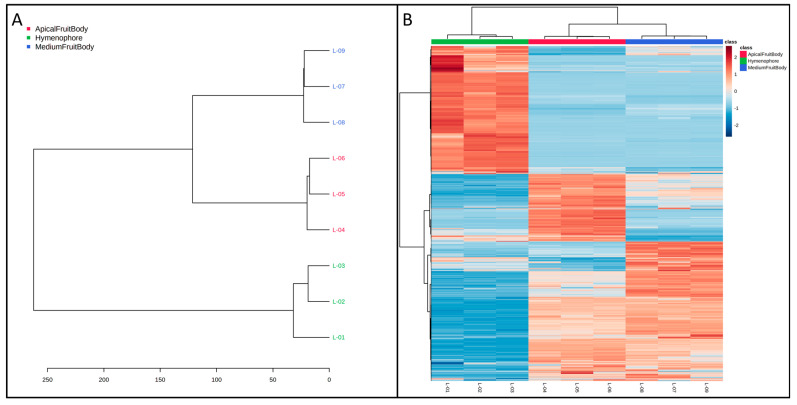
(**A**) Dendrogram and (**B**) heat map confirming that the samples were mainly divided into two clusters: hymenium and fruiting body. The latter was divided into the medium and apical clusters. Heat-map analysis shows that some characteristics were overexpressed only in the hymenophore, others only in the apical part of the fruiting body, and others only in the middle part of the fruiting body.

**Figure 3:**
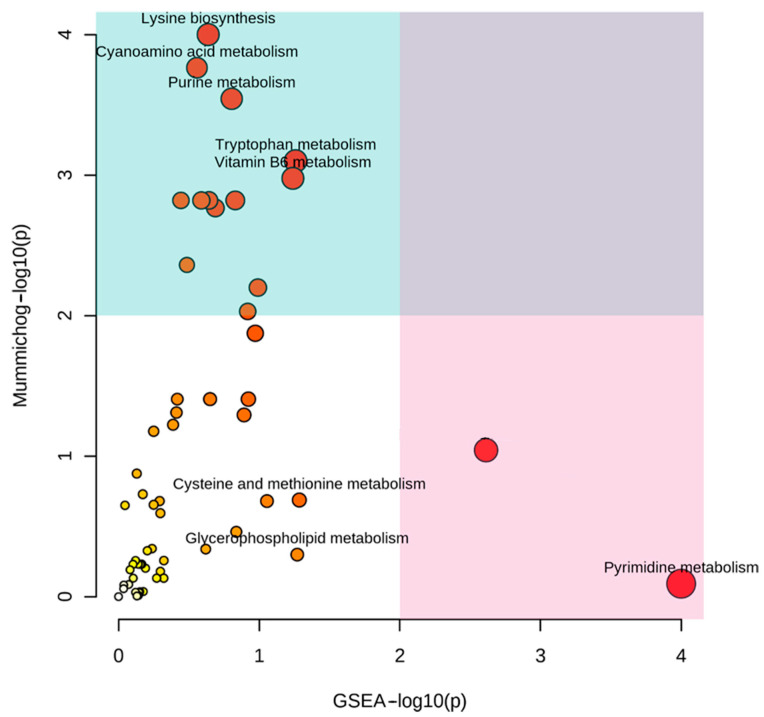
Statistically significant pathways active in the hymenophore.

RawData_Mz__RT_MetaboAnalyst.xlsx was uploaded to the MetaboAnalyst web platform to perform PCA, and heat-map, pathway, and functional analyses (Figure 1, Figure 2 and Figure 3). To perform PCA, the data matrix was treated with autoscaling and normalized with the median.

### 2.4. Cluster Analysis

The data, treated as previously described, were used to create the dendrogram (Figure 2A) and the heat maps (Figure 2B). The distance between the features was calculated with the Euclidean algorithm.

### 2.5. Functional Analysis

Both PCA and cluster analysis showed a clear separation between the hymenium and the fruiting body. Therefore, the data were subjected to functional analysis to search for significantly altered metabolic pathways between these two groups.

The data were loaded into the MetaboAnalyst functional analysis module, which performs a putative annotation of the features on the basis of *m*/*z* and RT values obtained from spectrometric analysis. A putative list of 505 annotated metabolites (AnnotatiMetaboAnalyst.csv) was used to determine the active metabolic pathways using the Mummichog and GSEA algorithms. The result sof this analysis are shown in Figure 3, where the abscissa and ordinate show the −log10 of the *p*-value calculated with the GSEA and Mummichog algorithms, respectively. The size of the circles represents the enrichment factor of each pathway; the color fades from yellow into red in proportion to the −log10 of the probability that a given pathway was active. The pathways that were statistically significantly active in the hymenium with respect to the fruiting body fell into the upper and right quadrants of the figure. A total of 60 metabolic pathways were identified; the complete data are in the table “Results_mummichog_integ_pathway_enrichment”.

Table 2 shows the most significant pathways (combined *p* value < 0.2).

### 2.6. Pathway Analysis of the Apical Part with Respect to the Middle Part of the Fruiting Body

The matrix showing the metabolites found with functional analysis was used to determine the metabolic differences between the apical and middle parts of the fruiting body, and statistical analyses show that they differed markedly from each other. The metabolic pathways that were overexpressed in the apical part compared to the middle part are listed in Table 3.

### 2.7. Extract Phenolic Composition and Antioxidant Activity 

Phenolic compounds are important phytochemicals whose content in plant extracts is generally determined with the Folin-Ciocalteu spectrophotometric and HPLC methods. Table 4 shows the values of the total phenol content (TPC) and antioxidant activity of the extracts of the three parts of F. officinalis (hymenium, apical, and median parts). The values for total phenoliv content ranged from 89.61 mg GAE/100 g in the median part to 116.12 mg GAE/100 g in the apical part of the mushroom.

The apical part (L4–L6) was richer in total phenols, which was confirmed with chromatographic analysis (Supplementary Material Tables S1 and S2, Figures S1–S4). Among the identified compounds, quercetin was the main phenolic compound.

The recent interest in phenolic compounds characterizing edible mushrooms has been due to their health-promoting properties, such as their antioxidant potential. In order to evaluate the antioxidant activity of extracts, three complementary spectrophotometric in vitro assays were carried out. ABTS and DPPH assays measure the ability of antioxidants to scavenge chromogen ABTS or chromogen DPPH free radicals, respectively, while the FRAP assay allows for evaluating the reducing capacity of the extracts. All these assays were compared with the Trolox standard, a water-soluble vitamin E analog.

In this paper, the highest values of ABTS (170.00 mg TE/g DW), DPPH (104.06 mg TE/g DW), and FRAP (198.00 mg TE/g DW) were found in apical part of F. officinalis. These results agree with the phenolic content; in fact, the lowest values of all spectrophotometric assays were found for the hymenium. A wide range of values of TPC and antioxidant activity was reported in a previous paper [22] that studied the optimization of the extraction of bioactives from Pleurotus ostreatus. TPC values ranged from 38.5 to 423.7 mg GAE/100 g and were all lower than those reported in this paper. Concerning antioxidant properties, Ianni et al. [22] reported a wide range for FRAP (6.0–70.0 mg TE/100 g value) and DPPH (8.7 to 172.0 mg TE/100 g) values, which are all lower than those obtained for F. officinalis, while ABTS values (110.7–1112.7 mg TE/100 g) were similar or lower, a comparison of the antioxidant assay results with those in the literature was sometimes not possible because the data of these assays were reported as EC_50_ or radical scavenging activity (%) [11,19,23,24,25]. A correlation study was also conducted considering all the extracts and all spectrophotometric parameters (Table 5). Good correlations were always obtained (R^2^ higher than 0.6874), and the best correlation values were found for TPC vs. FRAP (R^2^ = 0.9886) and DPPH vs. ABTS (R^2^ = 0.9755).

### 2.8. Antimicrobial Activity

In the present study, the results of the antimicrobial effect of *F. officinalis* were evaluated. The antimicrobial activity of *F. officinalis* Extracts L1, L4, and L7 against the tested bacterial, yeast, and dermatophytic strains is shown in Table 6, Table 7 and Table 8. All *F.* officinalis extracts showed antimicrobial activity in the concentration range of 1.95–200 µg/mL, but with a wide variability in terms of potency and selectivity (Table 6 and Table 7). The growth inhibition of the yeast strains (Table 7) showed no sensitivity to the L1, L4, and L7 *F. officinalis* extracts. Regarding the growth inhibition of dermatophytic isolates (Table 8), *T. tonsurans* (CCF 4834), *T. erinacei* (CCF 5930), *A. gypseum* (CCF 6261), *A. currei* (CCF 5207) and *A. insingulare* (CCF 5417) were resistant to *F. officinalis* Extract L1; the strongest inhibition was observed for *F. officinalis* Extract L4 against *T. tonsurans* with an MIC value of 19.57 µg/mL; *T. interdigitale* was sensitive to all tested extracts with an MIC range between 31.49 and 125.99 µg/mL.

With reference to bacteria (Table 6), the highest antimicrobial activity of *F. officinalis* extracts was observed in Extract L1 (MIC 1.53–<1.53) against *Bacillus subtilis* (PeruMycA 6), and Extracts L4 and L7 (MIC 3.86 and 7.71 µg/mL) against *Escherichia coli* (ATCC 10,536) with an MIC range of 3.86–79.37 μg/mL; *S. typhi* (PeruMycA 7) was the most resistant strain to *F. officinalis* extracts with an MIC range of 158.74–>200. There were only two cases in which there was the least sensibility for L1 against *Pseudomonas aeruginosa* (ATCC 15442) and *Staphylococcus aureus* (ATCC 6538). In this case, the extracts also showed a wide range of sensibility (MIC 7.71 (6.12–12.25)–158.74 (100–200) µg/mL). MIC values under 100 µg/mL were considered an indication of high antimicrobial activity [26]. The highest antimicrobial activity of L1 was observed against *B. subtilis* (MIC 1.53 µg/mL), L4 had a major affect towards *E. coli* (MIC 3.86 µg/mL), and the highest inhibition of L7 was again observed for *B. subtilis* (MIC 3.86 µg/mL). Collectively, Gram-negative bacterial strains (ATCC 10536 and 15442, PeruMycA 2, 3, and 6) were less sensitive to mushroom extracts than Gram-positive strains were.

Comparing antimicrobial activity results is not easy, as the used methodology to produce fungal extracts may vary widely, and susceptibility is not only species-specific, but even strain-specific [27].

Nevertheless, the reported MIC values in the present study could be compared to those reported for other Basidiomycota [27]. In a different study involving *Pleurotus ostreatus* [22], this was also true for the tested bacteria strains. *P. ostreatus* extracts showed major antibacterial activity towards Gram+ bacteria, and the highest MIC value (9.92 µg/mL) was observed against *B. subtilis* (PeruMyca 6), which was the same as *F. officinalis,* but the highest MIC concentration (<6.25 µg/mL) was towards *E. coli* (ATCC 10536).

In the same yeast strain, *P. ostreatus* had higher antimicrobial activity than that of *F. officinalis,* particularly against *C. albicans* (MIC 7.87 µg/mL; DBVPG 6379) [22]. The lowest sensitivity was observed for *C. tropicalis* (DBVPG 6184).

The MIC values of griseofulvin and fluconazole for strains *C. parapsilosis* (ATCC 22019) and *C. krusei* (ATCC 6258) were within the established ranges according to the CLSI M38 (CLSI 2018) and M38-Ed3 (CLSI 2017) protocols.

Overall, regarding the growth-inhibitory effects exerted by the *F. officinalis* extracts towards the selected pathogen microbial strains, consistent with the intrinsic biological activity of the extract, namely, antiradical properties, Extracts L4–L6 from the apical part were antimicrobially the most effective. This could partly be related to the phenolic compound content [28]. Although the MIC values were higher than those of the reference antimicrobial drugs, apical extracts were effective at <200 µg/mL concentrations, which are generally well-tolerated by human and murine cells [29]. Furthermore, MIC values < 100 μg/mL were considered an indication of high antimicrobial activity [26].

## 3. Materials and Methods

### 3.1. Chemical and Reagents

Folin–Ciocalteu’s reagent, 2,2′-azino-bis-(3-ethylbenzothiazoline-6-sulphonate) diammonium salt (ABTS), 6-hydroxy-2,5,7,8-tetramethyl-2-carboxylic acid (Trolox), 2, 2-diphenyl-1-picrylhydrazyl (DPPH), ferric chloride (FeCl_3_), sodium carbonate (Na_2_CO_3_), gallic acid (GA), and ethanol were purchased from Sigma-Aldrich (Milan, Italy). Mueller–Hinton broth (MHB), Rose Bengal Chloramphenicol Agar (RBCA), Malt Extract Agar (MEA), Tryptic Soy Agar (TSA), Sabouraud Dextrose Agar (SDA), RPMI (Roswell Park Memorial Institute) 1640 medium, and purity-grade organic solvents (Ethanol, and Dimethyl Sulfoxide) were purchased from Sigma (Sigma-Aldrich, Milan, Italy).

### 3.2. Mushroom Strain

The fruiting bodies of the *F. officinalis* strain (PeruMyc 3897) were collected from *Larix decidua* Mill. in Malga Campo (38080 Caderzone TN; 1970 m a.s.l.) in September 2021. The Vaucher specimen (height: 35 cm; length: 15 cm; thickness: 15 cm) was identified on the basis of macro–microscopic features and was deposited in the herbarium of the University of Perugia (Department of Chemistry, Biology and Biotechnology (DCBB), Perugia, Italy).

**Figure 4:**
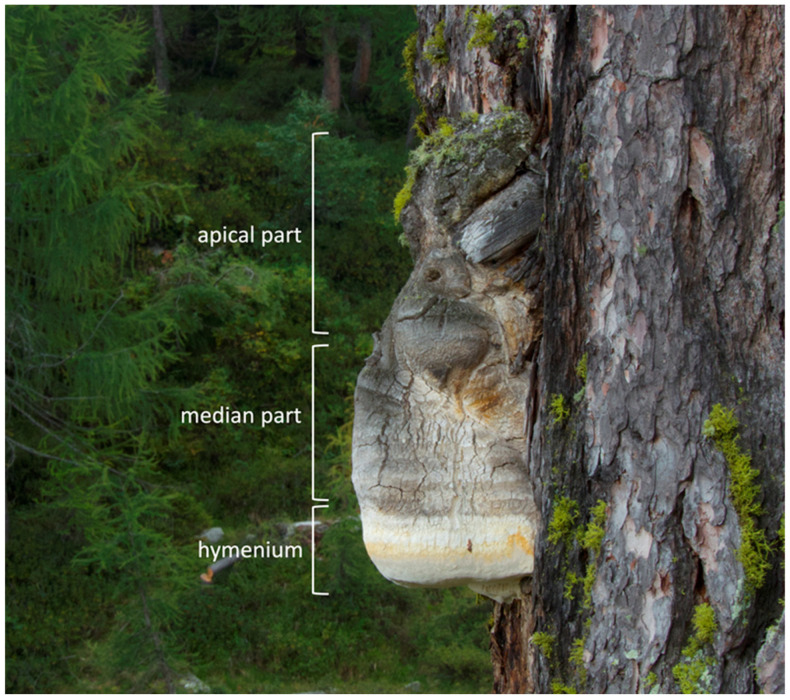
*F. officinalis* mushroom with the three investigated parts.

Figure 4 shows an *F. officinalis* mushroom with the three investigated parts.

### 3.3. Molecular Identification

Total genomic DNA was extracted from the fruiting body according to Angelini et al. [19]. The internal transcribed spacer (ITS) region was amplified using primer combination ITS1F/ITS4 according to Angelini et al. [19]. The thermocycler was programmed as follows: 1 cycle of denaturation at 95 °C for 2.5 min; 35 cycles of denaturation at 95 °C for 20 s, annealing at 55 °C for 20 s and extension at 72 °C for 45 s; 1 final extension cycle at 72 °C for 7 min. The electrophoresis of the polymerase chain reaction (PCR) amplicons was performed on 1.2% agarose gel. PCR products were purified using the ExoSap-IT PCR Cleanup reagent (Affymetrix UK Ltd., High Wycombe, UK) and then submitted for sequencing to Macrogen Europe (Amsterdam, The Netherlands). The resulting chromatogram was proofread, and the generated sequence was deposited in GenBank with access number OQ809067 (*F. officinalis* PeruMyc 3897).

### 3.4. Mushroom Extract Preparation

The mushroom materials were separated into 3 samples: the hymenium, median, and apical parts of the *F. officinalis* fruiting body. Afterwards, they were dried in a ventilated stove at 40 °C. The dried mushroom materials separated into hymenium, median, and apical parts of the fruiting body were finely ground and extracted in 50 mL of EtOH:water 7:3 (*v*/*v*) for 30 min under ultrasonic agitation. Each extract was prepared in triplicate (Samples L1–L9).

The resulting extracts were then filtered through Whatman GF/C filters (Sigma, Germany), and the solvent was evaporated under reduced pressure (40 °C, 218 mbar) using a rotary vacuum evaporator (Rotavapor R-100, Büchi, Switzerland). The residue was kept at −20 °C until further use (Table 9).

### 3.5. Spectrophotometric Assays

#### 3.5.1. Determination of Total Phenolic Content

The *F. officinalis* extract was mixed with 20% Na_2_CO_3_ solution and the Folin–Ciocalteu’s reagent, and the mixture was kept in the dark for 30 min before measuring the absorbance at 750 nm. The results, expressed as mg of gallic acid equivalents (GAE) per 100 g of dry weight (mg GAE/100g dw), were derived from the calibration curve of the gallic acid standard [22]. Table S3 shows the regression equation, linearity, and range of concentration of gallic acid.

#### 3.5.2. Determination of Antioxidant Activity

For the ABTS assay, radical cation ABTS^+^∙was prepared via the reaction of ABTS with potassium persulfate solutions after being kept in the dark at room temperature for 12 h. The obtained reagent was diluted with ethanol until 0.70 (±0.02) absorbance had been obtained at 734 nm. An aliquot of an ABTS^+^/ethanol solution was added to the extract, and the mixture was left in the dark for 6 min [22].

For the DPPH assay, the DPPH reagent (0.06 mM in ethanol) was added to the extract, and the mixture had been kept in the dark for 30 min before the absorbance was measured at 517 nm [22].

For the FRAP assay, the reagent, prepared by mixing a TPTZ solution with a FeCl_3_ solution and acetate buffer, was added to the sample extracts. The reaction mixture had been kept in the dark for 4 min before absorbance was measured at 593 nm [22].

The results of the antioxidant assays are expressed as mg Trolox equivalents (TE) per 100 g of dry weight (mg TE/100g dw) and were derived from a calibration curve of the Trolox standard (Table S3).

### 3.6. Untargeted LC–MS/MS-Based Metabolomics and Statistical Analysis

Untargeted LC/MS QTOF analysis was performed using a 1260 Infinity II LC System coupled with an Agilent 6530 Q-TOF spectrometer (Agilent Technologies, Santa Clara, CA USA). The LC consisted of a quaternary pump, a thermostatted column compartment, and an autosampler. Separation was conducted on an Agilent InfinityLab Poroshell 120 HILIC-Z, 2.1 × 150 mm, 2.7 µm at 25 °C, and 0.25 mL/min flow. The mobile phase consisted of a mixture of water (A) and water/ACN 15:85 (B)m with both containing a concentration of 10 mM ammonium acetate. The gradient was time 0–3 min, isocratic at A 2%, B 98%; time from 3 to 11 min: linear gradient to A 30%, B 70; time 11–12 min: linear gradient to A 60%, B 40%; time from 12 to 16 min: linear-gradient to A 95%, B 5%; time 16–18 min: isocratic at A 95%, B 5%; time 18 min: stop run.

Spectrometric data were acquired in the 40–1700 *m*/*z* range in both negative and positive polarity. The Agilent JetStream source was operated as follows: gas temperature (N_2_) 200 °C, drying gas 10 L/min, nebulizer 50 psi, sheath-gas temp: 300 °C at 12 L/min.

Raw data were processed using the MS-DIAL software (4.48) [30] to perform peak-picking, alignment, and peak integration. The MS signal threshold was set at 1000 counts. A data matrix was obtained that accurately reported the mass and area of each revealed peak in each analyzed sample.

Metabolites were putatively annotated and metabolic pathways were predicted using the mummichog algorithm [31] implemented in the ‘MS Peaks to Pathways’ module of Metaboanalyst 5.0 [32]. This considers any possible adducts and different ionic polarities, and classifies annotated peaks on the basis of the t-test. In this case, the list of putative compounds was mapped onto the KEGG library of *Saccaromices cerevisiae*. ANOVA and functional meta-analysis were also performed with MetaboAnalyst. For the statistical analysis, samples were normalized via the median, followed by Pareto scaling.

### 3.7. HPLC Determination of Phenolic Compounds

The extracts were analyzed for quantitative phenolic determination using a re-versed-phase HPLC-DAD in gradient elution mode [33]. The separation was conducted within 60 min of the chromatographic run, starting with the following separation conditions: 97% water with 0.1% formic acid, 3% methanol with 0.1% formic acid (Supplementary Table S1). The separation was performed on an Infinity lab Poroshell 120-SB reverse-phase column (C18, 150 × 4.6 mm i.d., 2.7 μm; Agilent, Santa Clara, CA, USA). Column temperature was set at 30 °C. The quantitative determination of phenolic compounds was performed via a DAD detector. The selected wavelengths are reported in Supplementary Table S2. The quantification was conducted through 7-point calibration curves with linearity coefficients (R^2^) > 0.999 in the concentration range of 2–140 μg/mL. All standards were purchased from Merck Life Science (Milan, Italy), and had ≥95% purity. The limits of detection were lower than 1 μg/mL for all assayed analytes. The area under the curve from the HPLC chromatograms was used to quantify the analyte concentrations in the extract [33].

### 3.8. Antimicrobial Test

#### Bacterial and Fungal Strains

The in vitro antimicrobial activity of the *F. officinalis* extracts (Samples L1–L9) was assessed against the following Gram– and Gram+ bacterial strains: *Escherichia coli* (ATCC 10536), *E. coli* (PeruMycA 2), *E. coli* (PeruMycA 3), *Bacillus cereus* (ATCC 12826), *Pseudomonas aeruginosa* (ATCC 15442), *B. subtilis* (PeruMyc 6), *Salmonella typhi* (PeruMyc 7), and *Staphylococcus aureus* (ATCC 6538). Furthermore, we performed antifungal assays on the same extracts against different yeast, dermatophytic, and fungal-pool species: *Candida albicans* (YEPGA 6183), *C. tropicalis* (YEPGA 6184), *C. albicans* (YEPGA 6379), *C. parapsilopsis* (YEPGA 6551), *Arthroderma curreyi* (CCF 5207), *A. gypseum* (CCF 6261), *A. insingulare* (CCF 5417), *A. quadrifidum* (CCF 5792), *Trichophyton mentagrophytes* (CCF 4823), *T. mentagrophytes* (CCF 5930), *T. rubrum* (CCF 4933), and *T. tonsurans* (CCF 4834).

The *Candida parapsilosis* (ATCC 22019) and *Candida krusei* (ATCC 6258) strains were used as quality controls in the broth dilution antifungal test [34].

### 3.9. Antibacterial Activity

The MICs of the *F. officinalis* samples were determined in sterile 96-well microplates using the broth microdilution method of the Clinical and Laboratory Standards Institute, M07-A10 [34]. MICs were determined using extract concentrations in the range of 1.562–200 µg/mL, derived from serial twofold dilutions in Mueller–Hinton Broth (MHB).

Ciprofloxacin (Sigma, Germany) was used in the range of 0.12–125^−1^ µg/mL as a control antibacterial agent [11].

For the preparation of the bacterial suspensions (inocula), three to five colonies of the bacterial strains used for the test were chosen from 24 h cultures on tryptic soy agar plates (TSA) and pregrown overnight in Mueller–Hinton broth (MHB) to reach a cell density of approximately 1–2 × 10^8^ CFU/mL in each tube.

This was confirmed with the plating of serial dilutions of the inoculum suspensions on Mueller–Hinton Agar (MHA). The setup included bacterial growth controls in wells containing 10 μL of the test inoculum and negative controls without a bacterial inoculum. MIC end points were determined after 18–20 h incubation in ambient air at 35 °C.

### 3.10. Antifungal Activity

Susceptibility testing against the yeasts and filamentous fungi was performed according to the CLSI M38 (CLSI 2018) and M38-Ed3 (CLSI 2017) protocols [34,35]. A Roswell Park Memorial Institute (RPMI) 1640 medium (Sigma) with L-glutamine, without sodium bicarbonate, and supplemented with 2% glucose (*w*/*v*), buffered with 0.165 mol/L morpholinepropanesulphonic acid (MOPS), pH 7.0, was used throughout the study.

The inoculum suspensions were prepared from 7-day-old cultures grown on Sabouraud Dextrose Agar (SDA; Difco) at 25 °C, and adjusted spectrophotometrically to optical densities that ranged from 0.09 to 0.11 (MacFarland standard).

Filamentous fungi (microconidia) and yeast inoculum suspensions were diluted to a ratio of 1:50 in RPMI 1640 to obtain twice the inoculum size, ranging from 0.2 to 0.4 × 10^4−5^ CFU/mL. This was further confirmed by plating the serial dilutions of the inoculum suspensions on SDA.

*F. officinalis* extracts had an MIC range of 1.56–200 µg/mL^−1^, fluconazole (Novartis, Basel, Switzerland) had an MIC range of 0.03–16 µg/mL^−1^, and griseofulvin (Sigma) had an MIC range of 0.03–8 µg/mL^−1^ [11].

MIC end points (μg/mL) were determined after 24 h (for yeasts) and 72 h (for dermatophytes) of incubation in ambient air at 30 °C (CLSI 2017, CLSI 2018). For the *Fomitopsis* extracts, ç MIC end points were defined as the lowest concentration that showed total growth inhibition.

ç MIC end points for fluconazole and griseofulvin were defined as the lowest concentration that inhibited 50% of the growth when compared with the growth control (CLSI 2017). Geometric means and MIC ranges were determined from the three biological replicates to allow for comparisons between the activities of the *F. officinalis* samples.

### 3.11. Statistical Analysis

The results of spectrophotometric assays (TPC, ABTS, DPPH, FRAP) are reported as the mean ± standard deviation (SD) of three replicates. Microsoft Excel 2016 (Microsoft Corporation, Redmond, WA, USA) was used for data analysis. Correlation analysis was performed with a linear regression model.

## 4. Conclusions

Due to technological developments, mass spectrometry matched with liquid chromatography could be largely employed in metabolomic studies with a broad perceived range, and advanced specificity and sensitivity. In the current study, this method was used to analyze the metabolic profiling of *F. officinalis* extracts (Samples L1, L4, and L7), showing satisfactory data quality. The present findings support more indepth investigations aimed at evaluating the influence of growth substrates on the antimicrobial and antioxidant properties of *F. officinalis*. Extracts from distinct parts of the fruiting body of *F. officinalis* revealed different concentrations of secondary metabolites, thus suggesting potential applications in the formulation of food supplements with biological properties, especially in terms of antioxidant and antimicrobial activities.

## Figures and Tables

**Table 1:** GenBank sequences and identity percentages with *Fomitopsis officinalis* strain (PeruMyc 3897) studied in this work.

Species	Sample ID	Country	Base Pair	Correspondence with Genbank Seq.	Identity Percentage	Accession No.
*F. officinalis*	MicUNIPV	Italy	597	*F. officinalis*	100	OL672134
*F. officinalis*	JFo3619	Poland	599	*F. officinalis*	100	MN534335
*F. officinalis*	LE-BIN 3560	Russia	605	*F. officinalis*	99.81	MG735354
*F. officinalis*	270279	Russia	553	*F. officinalis*	99.80	MF952886
*F. officinalis*	Stamets F04	USA	672	*F. officinalis*	99.62	EU854437

**Table 2:** Most significant pathways.

Pathway Name	Total_Size	Hits	Sig_Hits	Mummichog_Pvals	GSEA_Pvals	Combined_Pvals
Pyrimidine metabolism	17	17	4	0.937	0.01	0.05313
One carbon pool by folate	4	4	2	0.4849	0.04938	0.1133
Sphingolipid metabolism	4	4	2	0.4849	0.04938	0.1133
Tryptophan metabolism	12	12	7	0.1163	0.2347	0.1256
Lysine biosynthesis	15	15	9	0.06231	0.48	0.1349
Vitamin B6 metabolism	10	10	6	0.1267	0.24	0.1366
Purine metabolism	27	27	14	0.08555	0.396	0.1485
Cyano amino acid metabolism	7	7	5	0.07341	0.5263	0.1643

**Table 3:** Pathways overexpressed in the apical part compared to the middle part.

Pathway Name	Match Status	*p* Value	−LOG(*p*)	Holm *p*	FDR	Impact
Pyrimidine metabolism	17/34	1.13 × 10^−6^	5.946	7.47 × 10^−5^	7.47 × 10^−5^	0.56111
Tryptophan metabolism	12/30	2.78 × 10^−6^	5.5561	1.81 × 10^−4^	9.13 × 10^−5^	0.42636
Methane metabolism	13/23	6.43 × 10^−6^	5.1918	4.12 × 10^−4^	9.13 × 10^−5^	0.42562
Starch and sucrose metabolism	2/15	7.90 × 10^−6^	5.1023	4.98 × 10^−4^	9.13 × 10^−5^	0.4374
Folate biosynthesis	12/23	8.51 × 10^−6^	5.0703	5.27 × 10^−4^	9.13 × 10^−5^	0.53932
Galactose metabolism	6/17	9.64 × 10^−6^	5.0159	5.88 × 10^−4^	9.13 × 10^−5^	0.20513
Pantothenate and CoA biosynthesis	11/20	9.69 × 10^−6^	5.0138	5.88 × 10^−4^	9.13 × 10^−5^	0.48294
Phenylalanine, tyrosine, and tryptophan biosynthesis	15/21	1.98 × 10^−5^	4.7036	0.001167	1.63 × 10^−4^	0.62752
Valine, leucine, and isoleucine biosynthesis	14/20	2.76 × 10^−5^	4.5585	0.001603	1.96 × 10^−4^	0.61768
Glutathione metabolism	11/26	2.97 × 10^−5^	4.5273	0.001693	1.96 × 10^−4^	0.28415
Tyrosine metabolism	10/15	3.39 × 10^−5^	4.4697	0.001899	2.03 × 10^−4^	0
Glycerophospholipid metabolism	4/32	5.24 × 10^−5^	4.2808	0.002881	2.88 × 10^−4^	0.05355
Citrate (TCA) cycle	10/20	6.61 × 05	4.1796	0.003571	3.32 × 10^−4^	0.46979
Purine metabolism	29/62	8.47 × 10^−5^	4.0719	0.004491	3.32 × 10^−4^	0.44416
Pyruvate metabolism	11/23	8.48 × 10^−5^	4.0714	0.004491	3.32 × 10^−4^	0.52672
Cysteine and methionine metabolism	18/41	8.56 × 10^−5^	4.0673	0.004491	3.32 × 10^−4^	0.60695
Histidine metabolism	8/18	8.64 × 10^−5^	4.0636	0.004491	3.32 × 10^−4^	0.46939
beta-Alanine metabolism	4/11	9.04 × 10^−5^	4.0436	0.004491	3.32 × 10^−4^	0.5
Butanoate metabolism	9/14	1.02 × 10^−4^	3.993	0.004878	3.53 × 10^−4^	0.6
Sulfur metabolism	7/13	1.16 × 10^−4^	3.9364	0.005441	3.71 × 10^−4^	0.25975
Glycine, serine and threonine metabolism	16/32	1.18 × 10^−4^	3.9278	0.005441	3.71 × 10^−4^	0.56013
Biotin metabolism	3/13	1.39 × 10^−4^	3.8571	0.006253	4.17 × 10^−4^	0.21277
Lysine biosynthesis	8/16	1.48 × 10^−4^	3.831	0.006493	4.23 × 10^−4^	0.52557
Aminoacyl-tRNA biosynthesis	16/46	1.55 × 10^−4^	3.8106	0.006651	4.25 × 10^−4^	0.16667
Amino sugar and nucleotide sugar metabolism	4/24	1.86 × 10^−4^	3.7315	0.007793	4.90 × 10^−4^	0.07092
Glyoxylate and dicarboxylate metabolism	14/26	2.03 × 10^−4^	3.6926	0.00832	5.15 × 10^−4^	0.76807
Glycolysis/gluconeogenesis	6/24	2.17 × 10^−4^	3.6645	0.008661	5.29 × 10^−4^	0.24445
Pentose phosphate pathway	7/18	2.67 × 10^−4^	3.5737	0.010409	6.29 × 10^−4^	0.18245
Alanine, aspartate and glutamate metabolism	14/22	3.15 × 10^−4^	3.5012	0.011984	7.18 × 10^−4^	0.91008
Pentose and glucuronate interconversions	4/12	4.20 × 10^−4^	3.3763	0.015555	9.25 × 10^−4^	0.27273
Ubiquinone and other terpenoid-quinone biosynthesis	2/2	4.78 × 10^−4^	3.3209	0.017195	0.001017	0
Biosynthesis of unsaturated fatty acids	11/23	5.03 × 10^−4^	3.2988	0.017588	0.001037	0
Arginine biosynthesis	13/18	5.53 × 10^−4^	3.2576	0.018788	0.001105	0.69546
Sphingolipid metabolism	6/13	6.11 × 10^−4^	3.2143	0.020149	0.001185	0.76666
C5-Branched dibasic acid metabolism	3/4	7.42 × 10^−4^	3.1293	0.023758	0.001397	0
Arginine and proline metabolism	13/25	7.62 × 10^−4^	3.1181	0.023758	0.001397	0.59627
Vitamin B6 metabolism	4/11	7.89 × 10^−4^	3.1032	0.023758	0.001407	0.30769
Porphyrin and chlorophyll metabolism	12/23	8.48 × 10^−4^	3.0717	0.024588	0.001446	0.67455
Nicotinate and nicotinamide metabolism	8/12	8.73 × 10^−4^	3.0591	0.024588	0.001446	0.68086
Atrazine degradation	2/4	8.76 × 10^−4^	3.0574	0.024588	0.001446	0.5
One carbon pool by folate	5/8	0.001039	2.9835	0.027005	0.001672	0.63939
Fatty acid degradation	5/30	0.001234	2.9085	0.030861	0.00194	0.14049
Selenocompound metabolism	1/12	0.001829	2.7378	0.043894	0.002807	0.14286
Propanoate metabolism	2/19	0.001898	2.7218	0.043894	0.002846	0
Terpenoid backbone biosynthesis	7/16	0.002058	2.6865	0.045283	0.003019	0.41189
N-Glycan biosynthesis	3/31	0.002423	2.6156	0.050884	0.003477	0.07877
Steroid biosynthesis	7/30	0.002504	2.6014	0.050884	0.003516	0.41379
Valine, leucine and isoleucine degradation	6/18	0.002744	2.5617	0.052128	0.003772	0
Nitrogen metabolism	3/5	0.002913	2.5356	0.052437	0.003924	0
Fructose and mannose metabolism	2/14	0.003126	2.505	0.053139	0.004126	0
Lysine degradation	4/15	0.003409	2.4674	0.054543	0.004412	0.2
Fatty acid elongation	3/22	0.003517	2.4539	0.054543	0.004464	0
Thiamine metabolism	6/18	0.004475	2.3492	0.06265	0.005573	0.38119
Riboflavin metabolism	6/11	0.0061	2.2147	0.079301	0.007456	0.84849
Monobactam biosynthesis	2/4	0.007354	2.1335	0.088243	0.008824	0
Fatty acid biosynthesis	1/43	0.012753	1.8944	0.14028	0.01503	0
Phosphatidylinositol signaling system	2/26	0.014143	1.8494	0.14143	0.016094	0.08621
Inositol phosphate metabolism	2/22	0.014143	1.8494	0.14143	0.016094	0
Glycerolipid metabolism	1/14	0.017911	1.7469	0.14329	0.019943	0.07059
Taurine and hypotaurine metabolism	2/7	0.01813	1.7416	0.14329	0.019943	0
Sesquiterpenoid and triterpenoid biosynthesis	1/4	0.018708	1.728	0.14329	0.020241	0
Cyanoamino acid metabolism	5/8	0.035402	1.451	0.17701	0.037686	0
Carbapenem biosynthesis	1/3	0.10926	0.96153	0.43705	0.11447	0
Lipoic acid metabolism	1/6	0.15843	0.80017	0.47528	0.16338	0
Arachidonic acid metabolism	2/8	0.40465	0.39292	0.8093	0.41088	0
Synthesis and degradation of ketone bodies	1/3	0.55021	0.25947	0.8093	0.55021	0

**Table 4:** Values of total phenolic content and antioxidant activity (ABTS, DPPH, and FRAP) of the extracts (mean values ± SD, n = 3).

	TPC mg GAE/100 g	ABTS mg TE/100 g	DPPH mg TE/100 g	FRAP mg TE/100 g
L1–L3 hymenium	89.61 ± 8.70	144.39 ± 4.95	18.44 ± 0.28	119.97 ± 2.65
L4–L6 apical part	116.12 ± 3.45	170.00 ± 41.70	104.06 ± 5.44	198.00 ± 1.48
L7–L8 median part	92.02 ± 2.07	157.08 ± 12.60	72.60 ± 4.42	135.52 ± 1.79

TPC, total phenolic content; GAE, gallic acid equivalents; ABTS, 2,2′-azino-bis(3-ethylbenzothiazolin-6-sulfonic acid) diammonium salt; DPPH, 2,2-diphenyl-1-picrylhydrazyl; FRAP, ferric reducing antioxidant power; TE, Trolox equivalents.

**Table 5:** Correlation analysis (coefficient of determination, R^2^) among spectrophotometric parameters (TPC, DPPH, ABTS, and FRAP).

	TPC	ABTS	DPPH	FRAP
TPC	-	0.8216	0.6874	0.9886
ABTS	0.8216	-	0.9755	0.8956
DPPH	0.6874	0.9755	-	0.7817
FRAP	0.9886	0.8956	0.7817	-

**Table 6:** Minimal inhibitory concentrations (MICs) of *F. officinalis* samples against bacterial isolates.

	MIC (µg/mL) *							
	*Escherichia coli*	*Escherichia coli*	*Escherichia coli*	*Bacillus cereus*	*Pseudomonas aeruginosa*	*Bacillus subtilis*	*Salmonella typhi*	*Staphylococcus aureus*
Bacteria	(ATCC 10536)	(PeruMycA 2)	(PeruMycA 3)	(ATCC 12826)	(ATCC 15442)	(PeruMycA 6)	(PeruMycA 7)	(ATCC 6538)
Samples								
L1–L3	79.37 (50–100)	158.74 (100–200)	125.99 (100–200)	158.74 (100–200)	>200	1.53–<1.53	>200	>200
L4–L6	3.86 (3.06–6.12)	15.53 (12.25-25)	79.37 (50–100)	19.71 (12.25–25)	7.71 (6.12–12.25)	79.37 (50–100)	158.74 (100–200)	31.49 (25–50)
L7–L9	7.71 (6.12–12.25)	62.99 (50–100)	158.74 (100–200)	19.71 (12.25-25)	125.99 (100–200)	2.42 (1.53–3.06)	>200	39.68 (25–50)
Ciprofloxacin (µg/mL)	31.49 (25–50)	9.92 (6.25–12.5)	79.37 (50–100)	125.99 (100–200)	125.99 (100–200)	125.99 (100–200)	79.37 (50–100)	200–>200

* MIC values are reported as the geometric means of three independent replicates (n = 3). MIC range concentrations are reported within the brackets.

**Table 7:** Minimal inhibitory concentrations (MICs) of *F. officinalis* samples against yeast isolates.

	MIC (µg/mL) *			
	*Candida tropicalis*	*Candida albicans*	*Candida parapsilosis*	*Candida albicans*
Yeast Strain	(YEPGA 6184)	(YEPGA 6379)	(YEPGA 6551)	(YEPGA 6183)
Samples				
L1–L3	200–>200	200–>200	>200	>200
L4–L6	>200	>200	200–>200	>200
L7–L9	>200	>200	>200	>200
Fluconazole (µg/mL)	2	1	4	2

* MIC values are reported as the geometric means of three independent replicates (n = 3). MIC range concentrations are reported within the brackets.

**Table 8:** Minimal inhibitory concentrations (MICs) of *F. officinalis* samples against dermatophyticx isolates.

	MIC (µg/mL) *							
	*Trichophyton interdigitalis*	*Trichophyton tonsurans*	*Trichophyton rubrum*	*Arthroderma quadrifidum*	*Trichophyton erinacei*	*Arthroderma gypseum*	*Arthroderma currei*	*Arthroderma insingulare*
Dermatophyte	(CCF 4823)	(CCF 4834)	(CCF 4933)	(CCF 5792)	(CCF 5930)	(CCF 6261)	(CCF 5207)	(CCF 5417)
Samples								
L1–L3	125.99 (100–200)	200–>200	158.74 (100–200)	125.99 (100–200)	200–>200	200–>200	200–>200	200–>200
L4–L6	31.49 (25–50)	19.57 (12.25–50)	125.99 (100–200)	79.37 (50–100)	79.37 (50–100)	31.49 (25–50)	31.49 (25–50)	62.99 (50–100)
L7–L9	31.49 (25–50)	31.49 (25–50)	158.74 (100–200)	125.99 (100–200)	158.74 (100–200)	158.74 (100–200)	39.68 (25–50)	158.74 (100–200)
Griseofulvin (µg/mL)	2.52 (2–4)	0.198 (0.125–0.25)	1.26 (1–2)	>8	3.174 (2–4)	1.587 (1–2)	>8	>8

* MIC values are reported as the geometric means of three independent replicates (n = 3). MIC range concentrations are reported within the brackets.

**Table 9:** Preparation of *F. officinalis* samples.

Sample ID	Mushroom Sample	Dried Mushroom Weight (mg)	Added EtOH: H_2_O (mL)	Final Concentration (mg/mL)
L1	Hymenium	4700	50	94
L2	Hymenium	4700	50	94
L3	Hymenium	4700	50	94
L4	Apical part	4700	50	94
L5	Apical part	4700	50	94
L6	Apical part	4700	50	94
L7	Median part	4700	50	94
L8	Median part	4700	50	94
L9	Median part	4700	50	94

## Data Availability

The original data are available from the corresponding author.

## Supplementary Materials

The following supporting information about quantitative determination of phenolic compounds and antioxidant activity can be downloaded at: https://www.mdpi.com/article/10.3390/antibiotics12040766/s1; Table S1: Gradient elution condition of the HPLC–-DAD–-MS analyses; Table S2: Content in specialized metabolites; Table S3: Regression equation, linearity, range of concentration of standards (gallic acid for TPC, Trolox for ABTS, DPPH, and FRAP) used for the spectrophotometric assays; Chromatograms: Figures S1–S4.
