# Supplementary material for: Fomitopsis officinalis: Spatial (Pileus and Hymenophore) Metabolomic Variations Affect Functional Components and Biological Activities
Source: Antibiotics (Basel). 2023 Apr 16;12(4):766. doi: 10.3390/antibiotics12040766 (PMC10135358; doi:10.3390/antibiotics12040766)
Supplement: Supplementary file 1 [file antibiotics-12-00766-s001.zip › antibiotics-2310800-supplementary.pdf]

Fomitopsis officinalis: spatial (pileus and hymenophore) metabolomic variations affect functional components and biological activities

Giancarlo Angeles Flores [1,†], Gaia Cusumano [1,†], Federica Ianni [2], Francesca Blasi [2], Paola Angelini [1], Lina Cossignani [2,3*], Roberto Maria Pellegrino [1], Carla Emiliani [1], Roberto Venanzoni [1], Gokhan Zengin [4], Alessandra Acquaviva [5], Simonetta Cristina Di Simone [5], Maria Loreta Libero [5], Nilofar [5], Giustino Orlando [5], Luigi Menghini [5*], Claudio Ferrante [5]

[1] Department of Chemistry, Biology and Biotechnology, University of Perugia, 06122 Perugia, Italy; giancarlo.angelesflores@unich.it (G.A.F.); gaia.cusumano@studenti.unipg.it (G.C.); paola.angelini@unipg.it (P.A.); roberto.pellegrino@unipg.it (R.M.P.); carla.emiliani@unipg.it (C.E.); roberto.venanzoni@unipg.it (R.V.)
[2] Department of Pharmaceutical Sciences, University of Perugia, 06126 Perugia, Italy; federica.ianni@unipg.it (F.I.); francesca.blasi@unipg.it (F.B.); lina.cossignani@unipg.it (L.C.)
[3] Center for Perinatal and Reproductive Medicine, Santa Maria della Misericordia University Hospital, University of Perugia, 06132 Perugia, Italy
[4] Physiology and Biochemistry Research Laboratory, Department of Biology, Science Faculty, Selcuk University, 42130 Konya, Turkey; gokhanzengin@selcuk.edu.tr
[5] Botanic Garden "Giardino dei Semplici", Department of Pharmacy, "Gabriele d'Annunzio" University, Via dei Vestini 31, 66100 Chieti, Italy; alessandra.acquaviva@unich.it (A.A.); simonetta.disimone@unich.it (S.C.D.S.); maria.libero@unich.it (M.L.L.); nilofar.nilofar@unich.it (N.N.); giustino.orlando@unich.it (G.O.); luigi.menghini@unich.it (L.M.); claudio.ferrante@unich.it (C.F.)
* Correspondence: lina.cossignani@unipg.it (L.C.); luigi.menghini@unich.it (L.M.)
† These authors contributed equally to this work

Table S1. Gradient elution condition of HPLC–DAD–MS analyses.

TIME (min)	COMPOSITION A % (Water + Formic acid 0.1%)	COMPOSITION B % (Methanol + Formic acid 0.1%)	FLOW (mL/min)
1	97	3	0.6
5	77	23	0.6
12	73	27	0.6
18	57	43	0.6
25	52	48	0.6
32	50	50	0.6
34	50	50	0.6
37	35	65	0.6
40	5	95	0.6
47	10	90	0.6
48	10	90	0.6

Table S2. Content in specialized metabolites (µg/mL) of the tested *Fomitopsis officinalis* extracts. All identified phytochemicals have been identified through comparison with pure standards. Quantitative determination of the compounds was performed via DAD detector at 232–372 nm wavelength; nq: not quantified; nd: not detected.

	Components	Chemical Class	L1-L3 hymenium (µg/mg)	L4-L6 apical part (µg/mg)	L7-L8 median part (µg/mg)	Retention Time (Min)	Wavelenghts
1	Gallic acid	Benzoic acids	0.241±0.005	0.004±0.001	0.002±0.001	8.97	271
2	Caftaric acid	Hydroxycinnamic acids	0.298±0.013	0.035±0.004	0.047±0.002	12.92	310
3	Catechin	Flavanols	0.133±0.008	0.306±0.014	0.028±0.002	15.13	278
4	Gentisic acid	Benzoic acids	0.049±0.002	0.014±0.001	0.025±0.001	15.65	325
5	4-Hydroxybenzoic acid	Benzoic acids	nd	0.011±0.001	0.016±0.001	16.15	256
6	Loganic acid	Iridoids	nd	nq	0.007±0.001	16.94	232
7	Chlorogenic acid	Hydroxycinnamic acids	0.039±0.004	0.004±0.001	nd	17.41	325
8	Vanillic acid	Flavonol glycosides	0.028±0.001	0.010±0.001	0.012±0.001	18.77	257
9	Caffeic acid	Hydroxycinnamic acids	0.008±0.001	nd	nd	19.68	325
10	Epicatechin	Flavanols	nd	0.037±0.002	0.045±0.003	19.95	278
11	Syringic acid	Benzoic acids	nd	0.016±0.001	0.018±0.001	20.46	274
12	Syringaldehyde	Benzoic aldehydes	0.252±0.010	0.086±0.09	0.059±0.014	22.02	310
13	*p*-Coumaric acid	Hydroxycinnamic acids	0.081±0.001	0.340±0.014	0.022±0.002	23.24	310
14	*t*-Ferulic acid	Hydroxycinnamic acids	nq	nq	nq	24.41	315
15	Benzoic acid	Benzoic acids	0.052±0.003	0.016±0.005	0.028±0.011	26.71	275
16	Hyperoside	Flavonol glycosides	0.061±0.003	0.028±0.003	0.006±0.001	27.80	254
17	Rutin	Flavonol glycosides	nd	0.008±0.001	0.008±0.001	28.00	254
18	Isoquercetin	Flavonol glycosides	nq	nq	nq	28.17	340
19	Resveratrol	Stilbene	0.039±0.005	nd	0.041±0.007	28.50	310
20	Rosmarinic acid	Hydroxycinnamic acids	0.101±0.018	nd	nd	29.48	325
21	t-Cinnamic acid	Phenylpropanoids	0.028±0.002	nd	0.017±0.001	34.97	279
22	Quercetin	Flavonols	0.169±0.015	3.307±0.251	0.303±0.043	37.41	372
23	Naringenin	Flavanones	0.364±0.010	nd	nd	38.11	280
24	Hesperitin	Flavanones	1.880±0.041	0.035±0.007	0.050±0.012	40.31	280
25	Kaempferol	Flavanols	0.004±0.001	0.044±0.003	0.082±0.016	42.43	330
26	Carvacrol	Phenolic monoterpenes	0.015±0.003	0.036±0.002	0.086±0.013	45.08	275
27	Thymol	Phenolic monoterpenes	0.248±0.033	0.685±0.043	1.682±0.058	45.21	275
28	Flavone	Flavones	0.017±0.005	0.537±0.030	0.509±0.017	46.07	340
29	3-Hydroxyflavone	Flavones	0.081±0.007	0.620±0.030	0.696±0.012	46.33	340

Table S3. Regression equation, linearity, range of concentration of standards (gallic acid for TPC, Trolox for ABTS, DPPH, and FRAP) used for the spectrophotometric assays.

Assay	Calibration curve	R^2	Standard	Concentration
TPC	y = 11.98x − 0.0229	0.995	Gallic acid	0.0065-0.1 mg/mL
ABTS	y = - 1.6803x + 0.6872	0.999	Trolox	0.02-0.3 mg/mL
DPPH	y = - 1.6006x + 0.6393	0.999	Trolox	0.001-0.250 mg/mL
FRAP	y = 9.5883x – 0.0145	0.999	Trolox	0.0125-0.1 mg/mL

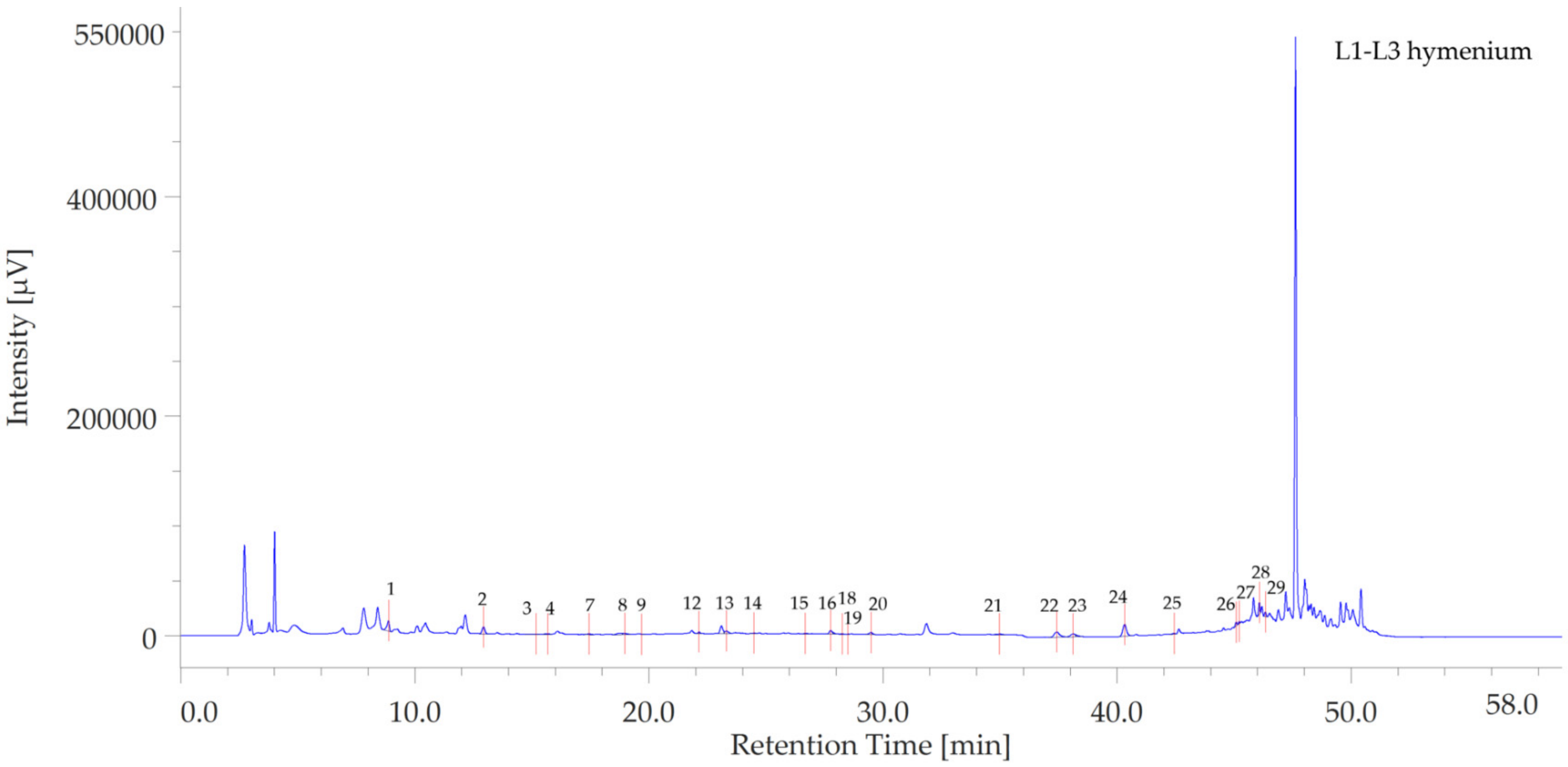

Figure S1. Chromatogram of hydroalcoholic extracts from the hymenium of *Fomitopsis officinalis*.

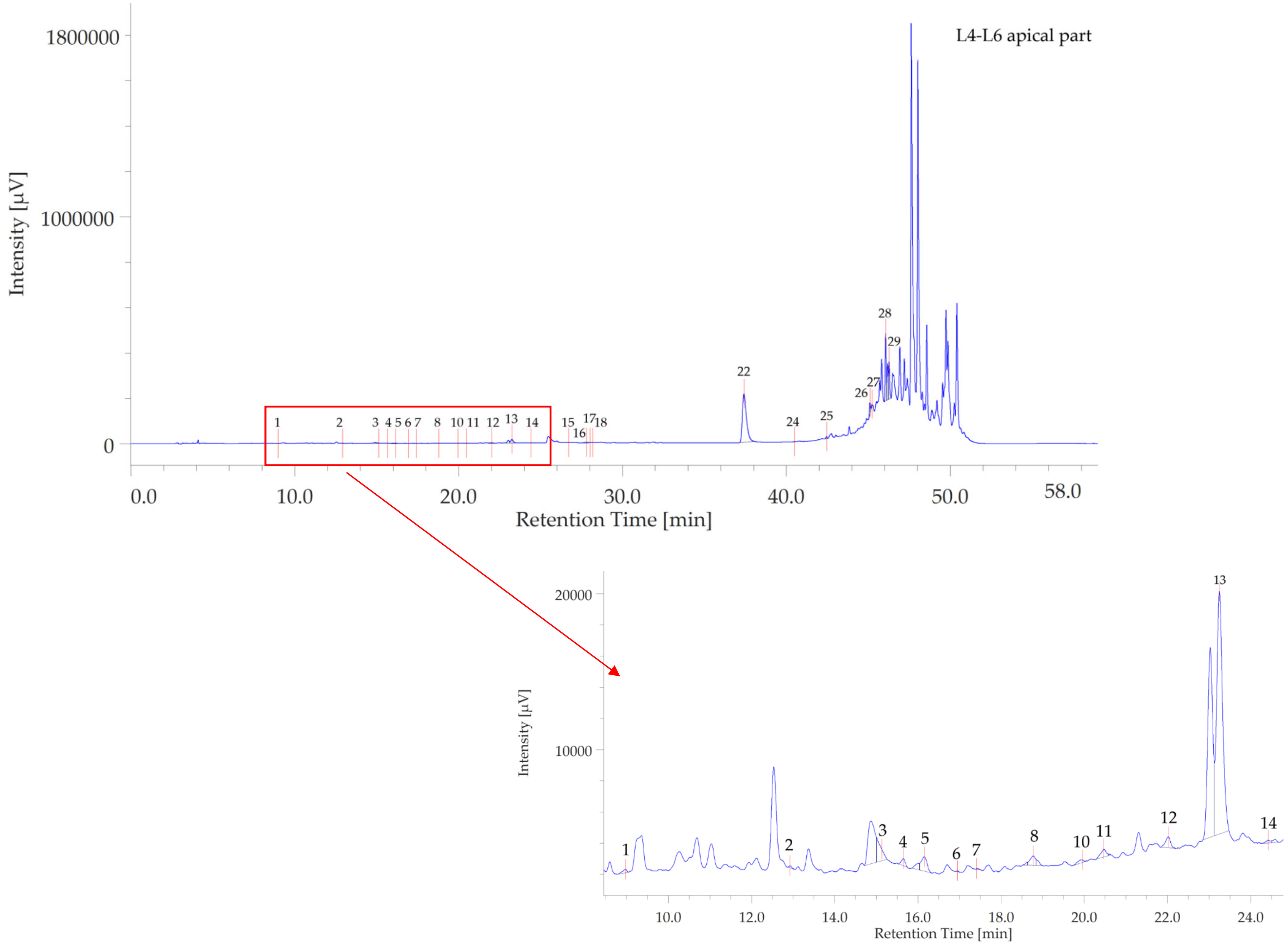

Figure S2. Chromatograms of hydroalcoholic extracts from the apical part of *Fomitopsis officinalis*.

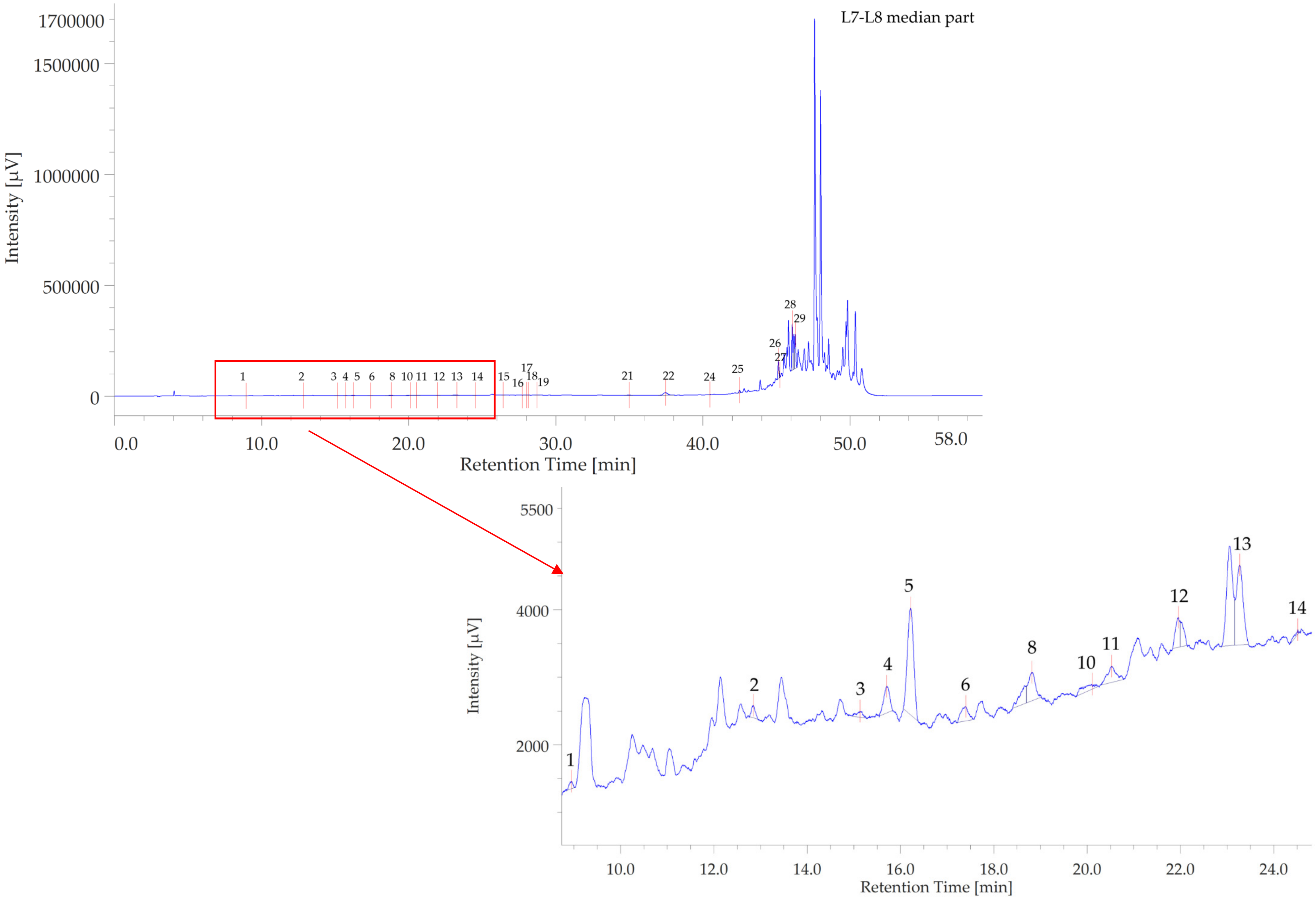

Figure S3. Chromatograms of hydroalcoholic extracts from the median part of *Fomitopsis officinalis*.

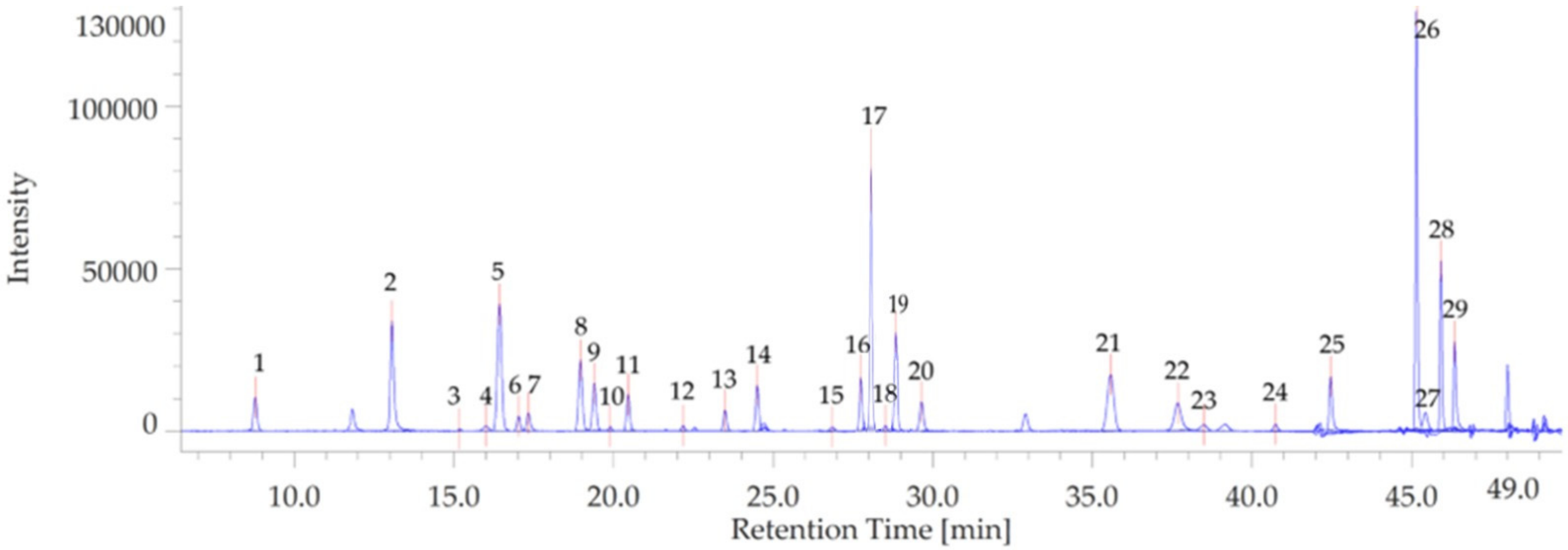

Figure S4. Chromatogram obtained from the standards.
